# Teachers’ Perceptions and Understanding of Children’s Fluid Intake

**DOI:** 10.3390/ijerph17114050

**Published:** 2020-06-05

**Authors:** Kristy Howells, Tara Coppinger

**Affiliations:** 1 Faculty of Education, Canterbury Christ Church University, Canterbury CT1 1QU, UK; 2 Department of Sport, Leisure & Childhood Studies, Cork Institute of Technology, T12 P928 Cork, Ireland; Tara.Coppinger@cit.ie

**Keywords:** fluid intake, teachers, children, school

## Abstract

No public health data exists on elementary teachers’ perceptions of both their own fluid intake and of their elementary school aged children’s fluid intake. A total of 271 (20 males, 251 females) teachers in developed areas of Australia, Belgium, England, Ireland, United Arab Emirates, and the United States of America completed an online questionnaire (Feb–Mar 2019) on: (i) their fluid intake, (ii) their perception and understanding of children’s fluid intake and (iii) barriers in the school day that they felt prevented school children consuming fluids. Overall, the data indicated that teachers consume considerably lower amounts than recommended themselves, but have a good awareness of children’s fluid intake and estimate children drink approximately half (1 litre (34% *n* = 93)) of what is recommended per day. The results were also similar to those reported by children previously. Yet, the data highlighted a lack of active encouragement of drinking water throughout the school day by teachers, with only 11% (*n* = 29) suggesting they actively encourage children to drink and 45% (*n* = 123) reporting no active encouragement at all. It is recommended as a public health measure that all school children consume an extra cup of water during lunch times in those schools where water intake was recognized as sub optimal. Furthermore, depending on weather conditions, a cup of water before, during and after Physical Education lessons should be encouraged by teachers. Water coolers or bottles may be used as a supplementary resource, provided that hygiene is maintained. From an educational perspective, more professional development needs to be provided to teachers on the importance of regular water consumption, and more time dedicated across the elementary curriculum to educational understanding of fluid consumption.

## 1. Introduction

Previous research has explored younger (4–5 years) and older (8 years 5 months SD ± 2 years 1 month) children’s knowledge and understanding of the consumption of fluids [1,2]. Findings from both studies reported that children struggle to understand, and know, how much, why and when to drink. In particular, children’s responses indicated that they did not understand fluid intake recommendations; they were unclear as to when they were allowed to drink across the school day and they found it difficult to articulate why drinking fluids was important [2]. Subsequently, it was recommended [1] that future research gauge the importance of the teacher and whether or not they are a key influencer in supporting children in their learning of how, why and when to drink.

Water should be encouraged as the main source of hydration for children for numerous health reasons; including the prevention of overweight and dental caries, yet, few countries report this being the case. Suboptimal cognitive function is repeatedly reported as a consequence of chronic dehydration in children, which can have adverse effects on learning. Children’s thirst responses are under developed compared to adults and they tend to experience a 45-min physiological delay in their thirst reflex and their ability to show any signs that they need to drink [3]. As a result, teachers may potentially find it difficult to know when to encourage children to drink. The lack of promotion of free drinking water could be exacerbating the issue with one study in Ireland in 2015 reporting that 40% of schools did not even provide free drinking water for students [4]. Another reason for the lack of promotion of fluid intake during school hours may be due to the limited nationwide campaigns that focus on increasing water intake across the school day. In the countries represented within the sample, there has been a limited number of specific drinking water campaigns targeted within schools and educational settings. For example, the last school drinking water campaign in England was ‘Water is Cool in School’ [5], which was launched over 13 years ago (2007) to promote water drinking in class time at primary schools, following findings that young children had inadequate fluid intake during school hours. In Australia, the focus by Government has been more on choices and encouraging all aged children to choose water as a healthy, non-sugary drink alternative in an attempt to change behavior and reduce children’s levels of overweight and to help prevent dental decay [6]. The ‘Drink Up’ campaign [7] in the United States of America (USA) was a collaborative campaign sponsored by supporters of tap, bottled, reusable, filtered and fountain companies to encourage everyone to drink water [8], whilst Northern Belgium’s focus was more on supporting the environment and reducing the number of plastic bottles being used through promoting the drinking of tap water [9]. In the United Arab Emirates (UAE), an annual campaign occurs which focuses on the health benefits of educating children to drink water to help support and maintain kidney health [10].

More recent campaigns have been undertaken globally that were beyond the representative countries in the sample. Thus, overall, this lack of attention being paid to the consumption of water in school could be having a negative effect on teacher’s promoting water intake in this setting. Furthermore, it is quite possible that new or recently qualified teachers have not themselves experienced the importance of fluid intake within the school day as learners, which could further impact on their perceived level of understanding, knowledge and behavior. Most recently in England, there has been a suggestion from the Department of Education [11] for school governors to encourage a healthy eating ethos and, for the first time ever, this ethos should include guidance on water consumption. Yet, no specific guidance has been given to governors as to how to support this. By investigating teachers’ understanding and knowledge of fluid intake, important findings could be obtained that could be regarded as being very relevant in the current educational climate.

It is also important for both children and teachers to understand the importance of adequate water consumption for health, as well as the consequences of lower than recommended intakes [12,13]. This is especially relevant given that previous research has found consuming school lunch to not be associated with water consumption among primary school aged children [14]. Unfortunately, there is very limited evidence supporting the need for increased availability of drinking water in schools to reduce sugar-sweetened beverage (SSB) consumption [15]. Nevertheless, their intake is associated with adverse health outcomes, particularly unhealthy weight gain and should therefore be discouraged [16,17]. The promotion of water intake for hydration and health purposes in school, rather than focusing on reducing SSB consumption, should be suggested as an alternative for policy makers, as free access to water during school hours may help promote children’s water consumption [18].

This study aims to investigate elementary school teachers’ perceptions and understanding of student’s fluid intake and what barriers in the school day prevent the encouragement of water consumption for children. Their own fluid intake, as well as how much they believed children drank within the school day, will also be investigated. This research examines teachers’ responses to similar questions posed to children on fluid intake, in order to offer a comparison to the teachers’ responses [1,2].

## 2. Materials and Methods

### 2.1. Participants

A total of 271 (251 females, 20 males) teachers from developed areas of Ireland, England, USA, UAE, Australia, Belgium and Australia participated in the study between February and March, 2019. There were 218 schools named by the participants, and 53 (36%) participants preferred not to identify their school. Schools ranged from a cross sectional representation of the type of schools, which included: church (faith), community, academies, public, private, voluntary-aided, voluntary controlled, international and European full-time schools. The focus was on the teachers’ perceptions and understanding, therefore no specific details of the pupils within the school were sought. The inclusion criteria for the teachers were that they taught within the primary and elementary age phase (children aged 5–13 years). Prior to study commencement, the lead researchers adapted and piloted the previously used questionnaire aimed at children [1] for use with teachers. The participant consent form was at the start of the online questionnaire and required the participants to agree to their answers being included in the data analysis, prior to commencing the survey. The participants undertook the online questionnaire via Google Forms and all the data were stored via password protected mechanisms, only accessible to the two lead researchers, and in line with GDPR. All participants remained anonymous and they were given the option to drop out at any time, without giving a reason. No personal details other than sex were asked, recorded and kept on file.

### 2.2. Procedure

**Figure 1:**
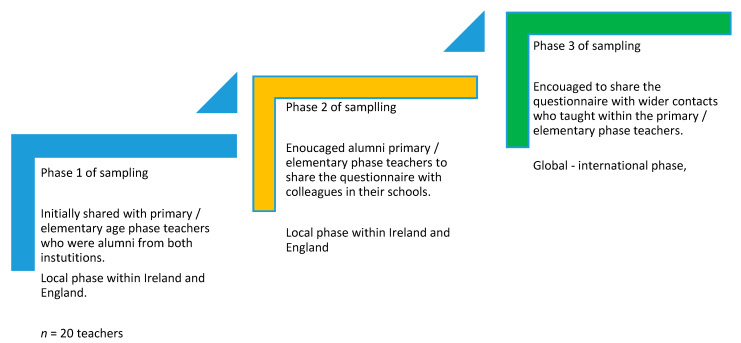
Snowball sampling technique.

Teachers were recruited via a virtual purposeful sampling technique [19] but this developed into a snowball sampling technique (see Figure 1).

The purposeful sampling commenced by the researchers targeting the alumni from both institutions (Canterbury Christ Church University, UK and Cork Institute of Technology, Ireland) (*n* = 20 teachers), and those teachers whose students had participated in the earlier children’s focused research [1,2]. Subsequently, the questionnaire was shared by these alumni to colleagues (causing the snowball and a change in the sampling technique), as the researchers first encouraged the alumni to share the questionnaire with colleagues in their schools and then to their wider contacts who taught within the primary/elementary phase. This enabled a wider network and geographical scope of interested participants to take part. Although similar characteristics remained to that of the original purposeful sample [20], the potential bias in the characteristics of the social networking online population is acknowledged. Without it, however, a smaller participant network of a targeted population of elementary school teachers, who were willing to share their thoughts and views, would have been captured. A short time frame was set for completion of the questionnaire (1 month) in an attempt to focus teachers on their current practice.

The questions within the questionnaire were based upon the previously validated questionnaire used by Coppinger and Howells [1]. The rating scale style questions were used to understand both the teachers’ own understanding and perceived fluid intake for themselves, as well as their understanding and knowledge of the children’s fluid intake that they currently teach. Open-ended style questions were also included to ascertain the teachers’ current school policy and ethos for fluid intake, barriers, as well as ideas and suggestions of how they could enhance fluid intake within their school. These types of questions were also used to obtain more in-depth answers from the participants.

### 2.3. Data Analysis

To avoid only part analysis of any questionnaire, any non-completed full sections of the questionnaire led to that entire questionnaire being completely withdrawn from the analysis. This removed 6 participants from the final analysis, to leave 271 participants in total. All responses were anonymized via an identification number and their subsequent results inputted into IBM SPSS Statistical for Windows (version 24.0, IBM Corp.: Armonk, NY, USA). Chi square statistical analysis were undertaken on all quantitative data and coding analysis completed on all qualitative responses. One multiple choice question focused on demographic information in order for direct comparison to be made across sex.

Ethical approval was granted from the ethic committees of both Canterbury Christ Church University (UK) and Cork Institute of Technology (Ireland) in March 2018.

## 3. Results

When asked how much they thought students drank in a school day, the most reported response by teachers was that children drink 1 litre a day (34% *n* = 93). However, when asked to estimate how much they thought students would have reported that they drink within a day, the majority (52% *n* = 142) estimated that most children would think they would finish one water bottle (500 mL).

### 3.1. When Do You Think Children Get Most Thirsty?

The two most popular times that the teachers’ indicated that the children get most thirsty was immediately after Physical Education (PE) lessons (39% *n* = 105) and after lunch times (30% *n* = 81) (Χ^2^, (6) = 2.745, *p* = 0.840). PE lessons are defined [21] as being part of the curriculum that focuses on physical fitness, health and hygiene. PE is learning through the physical means, learning through movement and moving to learn. None of the teachers reported that children seemed most thirsty at the start of the day. Some teachers did report that the children get most thirsty during lesson time (16% *n* = 43) but suggested this was due to boredom or avoidance strategies from the children not wanting to complete the lesson work.

### 3.2. What Activities in PE Do You Think Children Get Most Hot and Thirsty Participating in?

The most popular response by teachers as to what activities children are undertaking when they get hot and thirsty in PE was ‘running around’ (38% *n* = 103). Few felt that children got hot and thirsty in PE lessons when they were undertaking either team games or racket sports (0.3%, *n* = 1). However, 17% (*n* = 45) of teachers did not know when children would get the most hot and thirsty in their PE lessons (Χ^2^, (9) = 5.560, *p* = 0.783).

### 3.3. How Much Do Teachers Perceive They Themselves Drink on a Daily Basis?

**Figure 2:**
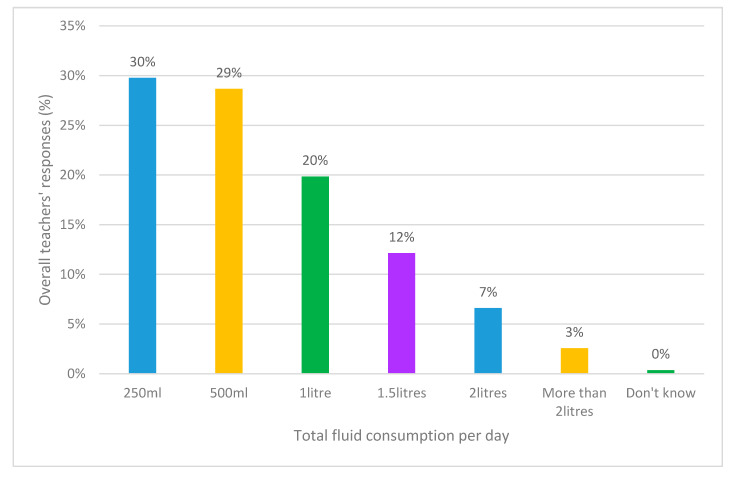
Teachers’ reported consumption of fluid per day.

The data found there were substantial contrasts to reported fluid intakes by teachers across the school day. The majority of teachers (91%, *n* = 246) reported drinking considerably lower amounts than what is recommended [13]. Of the gender adjusted water consumption, 20% (*n* = 5) of the male teachers consumed the recommended 3 litres of water per day, while 7% (*n* = 20) of female teachers consumed the recommended 2 litres of water per day. When analyzed as a whole group, the most popular responses were that they were drinking 250 mL (30% *n* = 81), 500 mL (29% *n* = 78) and 1 litre a day (20% *n* = 54) (see Figure 2).

In terms of what the teachers reported that they should be drinking, as opposed to what they did drink, the data did report a significant difference (Χ^2^, (6) = 20.458, *p* = 0.002). Only 1 (5%) of the male teachers reported that they should be drinking 3 bottles (2 litres), compared to 24% (*n* = 60) of female teachers; which meets the recommended intakes for females. A total of 20% (*n* = 4) of male teachers reported consuming the recommended more than 4 bottles (3 litres plus) a day, which meets the recommended intakes for males, compared to 8% (*n* = 20) of females [13].

Teachers were also asked how often they drank their own water in the classroom. Overall, 24% (*n* = 64) of teachers never or rarely had a drink or a water bottle within the classroom, thus limiting the teachers’ role modelling opportunities within the school day. The data also indicated a significant difference for sex (Χ^2^, (4) = 16.57, *p* = 0.002), with 45% (*n* = 9) of males stating they never (25%, *n* = 5) or rarely (20% *n* = 4) had a water bottle or drink in the classroom.

### 3.4. What Are the Barriers to Fluid Intake within School?

There were numerous parts of the school day that teachers reported children were not allowed to drink, with ‘during class time’ cited as the most popular reason (8% *n* = 23). Mass/assembly/prayers was next (6%, *n* = 18), followed by ‘when using electronic equipment’ such as ‘computers or Chromebooks’ (2%, *n* = 5). ‘Using specific subject specialist equipment,’ (3%, *n* = 8) which included equipment for welding, playing musical instruments, working with art resources and during Science lessons, were also specifically named by teachers as barriers to fluid intake. Not being allowed to drink during PE lessons (2% *n* = 5) or during break times/recess (1% *n* = 4) was also reported.

### 3.5. How Does Your School Currently Promote Fluid Consumption and where Are Children Allowed to Drink?

**Figure 3:**
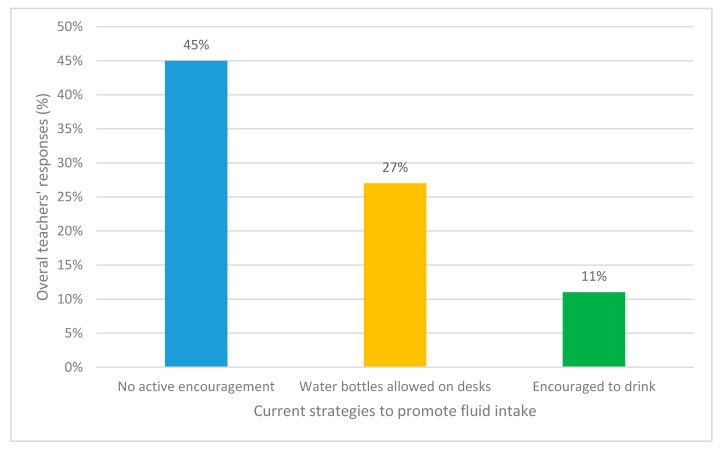
Teachers’ most popular responses for how their schools currently promote fluid consumption.

When teachers were asked how their school currently promotes fluid consumption, nearly half (45% *n* = 123) reported no active encouragement at all. Although, some did state that water bottles were allowed on the desks during lesson times. Some teachers suggested that they themselves would encourage children to drink throughout the day (see Figure 3). There were various parts of the school day that teachers reported children were allowed to drink. The most popular responses were ‘during or just after lunchtime,’ (49%, *n* = 133) ‘during or just after recess/break times’ (31%, *n* = 85) and ‘during snack time’ (18%, *n* = 51).

### 3.6. How Would You Like to Promote Water Intake within Your School?

**Figure 4:**
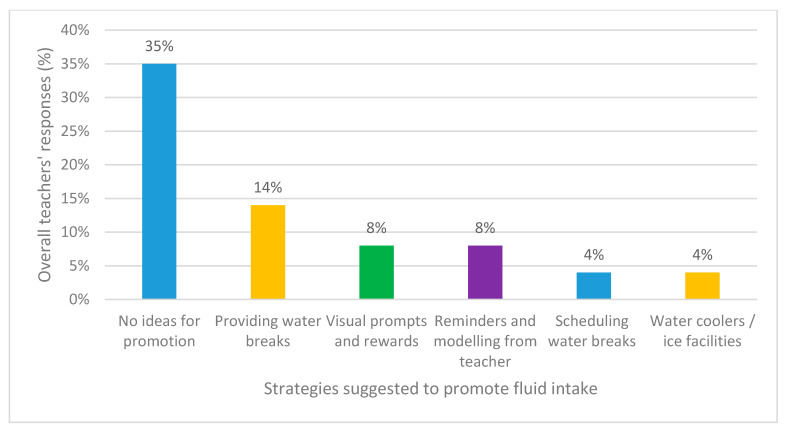
Teachers’ most popular responses for strateiges they would like to use to promote fluid intake within their school.

Teachers were asked to offer suggestions on how water intake could be promoted in their school setting. Over 1/3 (36%, *n* = 97) of all respondents did not have any ideas for how they would promote fluid intake within their school. The other most popular suggestions are illustrated in Figure 4.

## 4. Discussion

As children tend to spend half of their waking hours within an elementary school setting [22], investigating what teachers understand and know about fluid intake and the ways in which they are passing on this information to children through their learning, were central concepts for investigation within this study. It also aimed to explore the barriers that teachers face in encouraging fluid intake with elementary aged children, as well as their own understanding and perceptions of adult fluid intake. Examining responses to similar questions posed to children on fluid intake in order to offer a comparison to the teachers’ responses, was also undertaken. This study is original, multi-centered and of great importance to both the academic community and general public. It addresses a relevant topic that does not attract sufficient attention in the public discussion about health promotion nutrition.

The European Food Safety Authority [12] states that sufficient hydration is essential for maintaining children’s health, especially as they may not correctly replace fluid loss. Yet, according to Krecar et al. [23] and Sawka et al. [24], water is commonly ignored as a dietary essential and often overlooked, particularly in schools. This is supported by Howells [25] in her discussion of the new health education curriculum starting in England from September 2020, which states that drinking, and the impact of fluid intake, is often the forgotten part of food and diet for elementary aged children. Edmonds and Jeffes [26] found that children had higher levels of happiness when they had greater access to water and when fluid intake was promoted by teachers within an educational setting, indicating the importance of fluids and fluid intake not just for health but also for children’s overall wellbeing. Edmonds and Burford [27] also found children experienced a 10% improvement in cognition when they maintained good fluid intake.

For children aged 4–8 years (lower elementary), both boys and girls should be consuming 1.6 litres per day, of which 1.28 litres should be from fluids. Older children (9–13 year olds; upper elementary) have slightly higher requirements. Boys are encouraged to consume 2.1 litres, of which 1.68 litres should be from fluids alone, compared to girls who are recommended to consume 1.9 litres, of which 1.52 litres should come from fluids alone [12].

### 4.1. Do Teachers Know and Understand How Much Children Should be Drinking during the School Day?

Data in this research indicated that teachers thought that children drank between 500 mL and 1 litre a school day, yet these predicted levels were still lower than the daily amounts as recommended by the guidelines [12]. Although the question focused on drinking during the school day and not the whole day, considering that children spend half their waking hours in school, it could be suggested that schools should be responsible for only half the recommended drinking daily guidelines (640 mL for children aged 4–8 years; 840 mL for boys aged 9–13 years and 760 mL for girls aged 9–13 years, respectively) [12]. The results were similar to those reported by children themselves in previous research [1,2]. Although direct relationships cannot be determined from this study, this finding suggests that teachers have a good awareness of school children’s fluid intake across the school day. The fact that teachers reported some children to want a drink at the start of the day could be an indication of poor drinking habits prior to school. Given that skipping breakfast is common in some schoolchildren [28], this behavior could also be impacting their fluid intake; potentially meaning that children start school without having consumed any fluids for over 12 h. Teachers could potentially raise awareness with both children and parents/carers to help them encourage children to continue to drink at home and when they first arrive at school in the morning. By raising awareness within the educational and home setting, the combined efforts could help children maintain good levels of hydration to promote both their physical and mental wellbeing.

### 4.2. Do Teachers Know and Understand When Children Get Hot and Thirsty during PE?

The researchers concluded from the data that teachers were aware of when children get hot and thirsty during PE, and reported similar results to previous research [2] in which children reported running as the most reported activity for getting hot and thirsty. Within elementary and primary phase educational settings, it is important to note that the children have the same teacher for all subject disciplines; children do not move from teacher to teacher and therefore it is important for this educational context to focus on what happens within PE lessons. The children’s responses [2] also commonly reported ball and team games as times when they got hot and thirsty during PE, but this type of activity was the least reported by teachers in this study. This mismatch in identification for places that make children thirsty may mean that teachers do not promote drink breaks during team games, which may be putting these children at increased risk of poor hydration status, especially as team games tend to be of a moderate to vigorous intensity. Allowing children to drink before, as part of, and after, PE lessons is integral for ensuring children are able to replace their fluid loss correctly and ensure their overall wellbeing [26]. It is recommended as a public health measure therefore that depending on weather conditions, a cup of water before, during and after PE lessons, be encouraged by teachers.

### 4.3. Do Teachers Know and Understand How Much They Themselves Should be Drinking?

This question was asked to consider if the teachers themselves could be role modelling for the children during the school day, and acting as key influencers on fluid consumption. The researchers concluded from the data that the teachers were consuming significantly lower amounts of fluids (45% of males were drinking 2 litres less and 55% of females were drinking 1.5 litres or less, respectively) than recommended (2.5 litres for males, 2 litres for females, respectively) [13]. Although this does not account for fluid consumption intakes after school hours, it suggests that the potential to consume a substantial amount of their fluid requirements during their daily working hours is being missed. The fact that males were less likely to use water bottles within the classroom setting than females, (Χ^2^, (4) = 16.57, *p* = 0.002) could also be exacerbating the issue. This underestimation and limited role modelling by male teachers, in particular, would suggest a need for sex-specific support in fluid intake recommendations and educational material aimed at teachers. By supporting teachers to remain hydrated within the school day, could also help them to maintain higher levels of cognition [29] in their own teaching delivery.

### 4.4. Barriers for Promoting Fluid Intake?

Although it was previously reported [30] that increased fluid intake can cause an increased need to urinate, this disruption to teaching and learning was not reported in the current study. There were numerous occasions during the school day that teachers reported children were not allowed to drink, which was beyond specific subject areas that had been previously named by children in earlier research [1]. The most popular response was during class time (8% *n* = 23). Other times of the day that were reported as places not allowed to drink were mirrored in Coppinger and Howells [1] children’s responses. These included during PE lessons (2% *n* = 5) and at break times/recess (1% *n* = 4). The absence of fluid intake during these two times of the school day could inhibit children from learning to associate the importance of fluid consumption during moderate to vigorous intensity activity. This is of particular importance given that they have a reduced thirst reflex compared to adults [3]. It is recommended that professional development incorporates fluid education in order for teachers to understand and better implement drink breaks within educational settings; particularly at times of increased intensity activity.

### 4.5. Solutions for Promoting Fluid Intake?

Teachers are in a prime position to influence child health, but previous research found that training about health varies largely between teacher-training institutions in the UK [31]. In order for public health messages in educational settings to be successful, teachers therefore need to be provided with the knowledge and skills to realize their potential as health promoters, which this study looked to explore. Close to half (45% *n* = 123) of teachers reported that their schools did not have any active encouragement of fluid intake for students. This links to Williamson and Howells’ [2] research that found teachers were not regarded as the key influencers for child water consumption. Multiple suggestions were provided as to how fluid intake could be promoted by teachers; the most popular being to have access to water bottles at all times and/or by providing water bottles (14% *n* = 38). Yet, hygiene was not identified as an important precautionary measure of water bottle usage by any teacher. Reusable water bottles need to be regularly cleaned and disinfected to prevent water stagnation and the multiplication of water borne pathogens [32]. It is proposed that alongside the encouragement of greater water intake in schools that more time within the curriculum be dedicated to educate primary phased teachers and their students on the importance of water bottle hygiene. 

Concurring with recommendations from Williamson and Howells [2], a small number of responses suggested having water coolers and ice facilities, instead of fountains, to help keep water cold, which they felt would help encourage children to drink. Teacher-orientated recommendations were high in the suggestions and included reminders, setting scheduled water breaks, modelling themselves as an active role model, visual prompts/posters, setting rewards and challenges and the use of charts. Fascinatingly, none of the teachers suggested that they themselves needed educating on how much children should be drinking or on how they should be encouraging the children to drink.

## 5. Limitations

The study is limited due to it only providing a snapshot of cross-sectional data from teachers across the globe. It did not obtain measurements of the definite water intake of the participants and recommends future research to undertake a longitudinal study that measures actual teacher and student fluid intake, over time. Furthermore, due to there being a paucity of academic literature available detailing the effectiveness of water initiatives in each mentioned country in more detail in the study, the authors had to resort on occasion to available data from the internet, which could have been inaccurate and out of date. Future research should look to collaborate researchers from across the globe and aim to evaluate the effectiveness of any hydration initiatives aimed at school going children in their named country.

The data were analyzed for comparison by sex, as males and females have different adult daily recommended fluid intakes [13]. The lower male responses reflected in this study (7% *n* = 20 males, 93% *n* = 251 females) is acknowledged as a limitation but the study sample represented the sex bias that already exists in elementary schools, as teachers are predominantly female within this type of educational setting. The National Center for Education Statistics [33,34] reported the characteristics of public-school elementary teachers and identified males to consistently be less represented across numerous academic years, with staff being 11% male and 89% female in 2015–2016 and 11.4% males and 88.5% females, respectively, in 2017–2018.

Due to the virtual purposeful sampling technique [19] that snowballed into a wider geographical participant network and reach than was first anticipated when designing and implementing the online data collection tool, there was not the depth of participants from each country to allow for comparison across countries. Further purposeful sampling that is country specific could yield a cross country analysis, as well as examine any differences in initiatives implemented in temperate versus tropical regions.

## 6. Conclusions

Schools are a key place for children to form both life-long and life-wide habits and teachers can play a key role in influencing children’s understanding of the importance of correct fluid consumption. Previous research has already recommended that primary schools promote water drinking in class, but this study found few initiatives being implemented in schools. Policy makers need to address this gap if teachers are to be encouraged and supported to role model, enable and encourage children to drink at key moments in the school day. By doing so, children can then begin to recognize the importance of maintaining hydration during school hours. More longitudinal research that also investigates the actual water intakes of teachers and their students is also needed to inform future intervention strategies. By dedicating more time within the curriculum to the fluid consumption via several aspects of elementary curriculum including: health education; during PE, as well as science education, the educational understanding of fluid intake could also be delivered and emphasized.
